# Brief Interventions Influence the Quantity and Quality of Caregiver-Child Conversations in an Everyday Context

**DOI:** 10.3389/fpsyg.2021.645788

**Published:** 2021-06-16

**Authors:** Apoorva Shivaram, Yaritza Chavez, Erin Anderson, Autumn Fritz, Ryleigh Jackson, Louisa Edwards, Shelley Powers, Melissa Libertus, Susan Hespos

**Affiliations:** ^1^Department of Psychology, Northwestern University, Evanston, IL, United States; ^2^Department of Applied Statistics, University of Virginia, Charlottesville, VA, United States; ^3^Department of Psychology, Learning Research and Development Center, University of Pittsburgh, Pittsburgh, PA, United States

**Keywords:** cognitive development, informal learning, brief interventions, food pantry, caregiver-child conversations

## Abstract

Reading and arithmetic are difficult cognitive feats for children to master and youth from low-income communities are often less “school ready” in terms of letter and number recognition skills (Lee and Burkam, 2002). One way to prepare children for school is by encouraging caregivers to engage children in conversations about academically-relevant concepts by using numbers, recognizing shapes, and naming colors (Levine et al., 2010; Fisher et al., 2013). Previous research shows that caregiver-child conversations about these topics rarely take place in everyday contexts (Hassinger-Das et al., 2018), but interventions designed to encourage such conversations, like displaying signs in a grocery store, have resulted in significant increases in caregiver-child conversations (Ridge et al., 2015; Hanner et al., 2019). We investigated whether a similar brief intervention could change caregiver-child conversations in an everyday context. We observed 212 families in a volunteer-run facility where people who are food-insecure can select food from available donations. Volunteers greet all the clients as they pass through the aisles, offer food, and restock the shelves as needed. About 25% of the clients have children with them and our data consist of observations of the caregiver-child conversations with 2- to 10-year-old children. Half of the observation days consisted of a baseline condition in which the quantity and quality of caregiver-child conversation was observed as the client went through aisles where no signs were displayed, and volunteers merely greeted the clients. The other half of the observation days consisted of a brief intervention where signs were displayed (signs-up condition), where, volunteers greeted the clients and pointed out that there were signs displayed to entertain the children if they were interested. In addition, there was a within-subject manipulation for the intervention condition where each family interacted with two different categories of signs. Half of the signs had academically-relevant content and the other half had non-academically-relevant content. The results demonstrate that the brief intervention used in the signs-up condition increases the *quantity* of conversation between a caregiver and child. In addition, signs with academically-relevant content increases the *quality* of the conversation. These findings provide further evidence that brief interventions in an everyday context can change the caregiver-child conversation. Specifically, signs with academically-relevant content may promote school readiness.

## Introduction

Reading and arithmetic are uniquely human abilities that typically take several years of formal training in school to acquire (Duncan et al., 2007). Children who practice academic skills before the start of formal education have an advantage that is evident at the start of kindergarten, and this advantage continues to grow throughout elementary school (Lee and Burkam, 2002; Gibson et al., 2020; Susperreguy et al., 2020). One of the ways children learn how to read and do math outside of formal schooling is by being active learners and engaging with their environment, particularly within a social context (Piaget, 1954; Vygotsky, 1962, 1978; Tomasello et al., 2005). School-aged children spend less than 20% of their waking hours in formal educational settings (LIFE Center, 2005). As a result, children can develop academic skills through conversations with caregivers who may be particularly well-suited to tailor the conversational content to the individual child and their current context. Caregivers who produce higher amounts of child-directed speech tend to have children with stronger oral language skills (Huttenlocher et al., 1991; Hart and Risley, 1995; Hoff, 2003). Consequently, our first goal in this paper was to create situations in everyday contexts that could increase the *quantity* of conversations between a caregiver and child.

Children who discuss literacy and mathematics with their caregivers tend to have better academic and cognitive outcomes (Gunderson and Levine, 2011; Pruden et al., 2011; Sheridan et al., 2011; Susperreguy et al., 2020). Learning about academically-relevant concepts can be promoted in the home environment. Research indicates that an increase in caregiver-child early math talk is associated with better outcomes on children’s future math skills (Lombardi and Dearing, 2020; Son and Hur, 2020). Specifically, Gunderson and Levine (2011) found that children’s future understanding of cardinality (the number of items in a set) was best predicted by parent number talk using objects that were physically present in their immediate environment. Similarly, early spatial language such as naming shapes and colors also predicts the amount of spatial language that children produce. Shape and color talk in the home is indicative of later performance on spatial cognition tasks (Pruden et al., 2011), which has been linked to early mathematics performance (Mix and Cheng, 2012), STEM success (Wai et al., 2010), and school readiness (Verdine et al., 2014a). However, a recent meta-analysis by Anderson et al. (2021) reveals that definitions of conversational quality vary from study to study. In this paper, we define *quality* of conversation as variation in the different topics discussed with respect to number, color and shape talk. Our second goal was to test whether specific categories of questions were more effective than others in encouraging caregivers to engage in conversations about academically-relevant concepts like numbers, colors, and shapes in contrast to a more general language condition that consisted of non-academically-relevant content like questions that required one-word answers (e.g., how old are you?) or pronouncements (e.g., Everywhere you go, talk about what you see!). More broadly, our goal was to measure the *quality* of caregiver-child conversations in an everyday environment.

Despite the importance of integrating number, color and shape talk into conversations with children, there is wide variation in how much of the conversation between caregivers and children consist of these crucial topics (Levine et al., 2010; Gunderson and Levine, 2011; Pruden et al., 2011; Fisher et al., 2013; Resnick et al., 2016). There is growing evidence that children from lower-income families lag behind their peers from mid- and high-socioeconomic status (SES) families in terms of mathematical knowledge and that there is wide variability in the amount of caregiver-child math talk in their informal learning environments (Starkey et al., 2004; Ramani et al., 2015; Son and Hur, 2020). Similar differences are also found in the domain of color and shape talk, where lower-income families use significantly fewer spatial words during conversations compared to their higher-income peers (Bower et al., 2020; Verdine et al., 2014b). However, several studies have demonstrated that brief interventions can improve conversations between caregivers and children from lower-income families, particularly within informal learning environments such as grocery stores, libraries, bus stops, or at home (Starkey and Klein, 2000; Siegler and Ramani, 2008; Ridge et al., 2015; Hassinger-Das et al., 2020b). Our third goal was to test this kind of short-term intervention, to determine whether there is flexibility in how a family responds to these interventions based on the contents of the signage. Specifically, are individual families equally likely to engage in academically-relevant as well as non-academically-relevant conversations? We predict they will be.

Previous work provides evidence that a brief intervention of displaying signage in an everyday context of a grocery store can change the conversation between caregivers and children (Ridge et al., 2015; Hanner et al., 2019). Ridge et al. (2015) displayed signs in grocery stores located in low- and middle-SES neighborhoods and observed families’ conversations. These signs had questions like “Where does milk come from?” and “What is your favorite vegetable?” The authors found that for the grocery store in the low-SES neighborhood, the signs increased both *quantity* and *quality* of caregiver-child conversation compared to a baseline when there were no signs displayed. However, in the mid-SES neighborhood, there were no differences in conversations across the two conditions, likely because the interaction between caregivers and children was already high.

Hanner et al. (2019) replicated and extended Ridge et al.’s (2015) findings by focusing on math talk. They tested three conditions: math signs, general language signs, and a baseline with no signs. The math-sign condition encouraged caregivers to ask their children questions about numbers and math, such as “How many glasses of milk do you drink in a day/week?” The general language signs condition served as a control to ensure that any observed differences in math talk were a result of math-related prompts and not merely a result of posting signs. This condition had questions that were similar to those from Ridge et al. (2015) such as “Where does milk come from?” or “Why is milk good to drink?” The results demonstrated that the math signs were associated with significantly more math talk than the other two conditions. These math signs elicited more questions and conversations about principles of cardinality, counting, and calculation from caregivers and children compared to the general language and baseline conditions. Taken together, Ridge et al. (2015) and Hanner et al. (2019) show that brief interventions in an everyday context can change caregiver-child conversations in ways that may promote school readiness.

**FIGURE 1:**
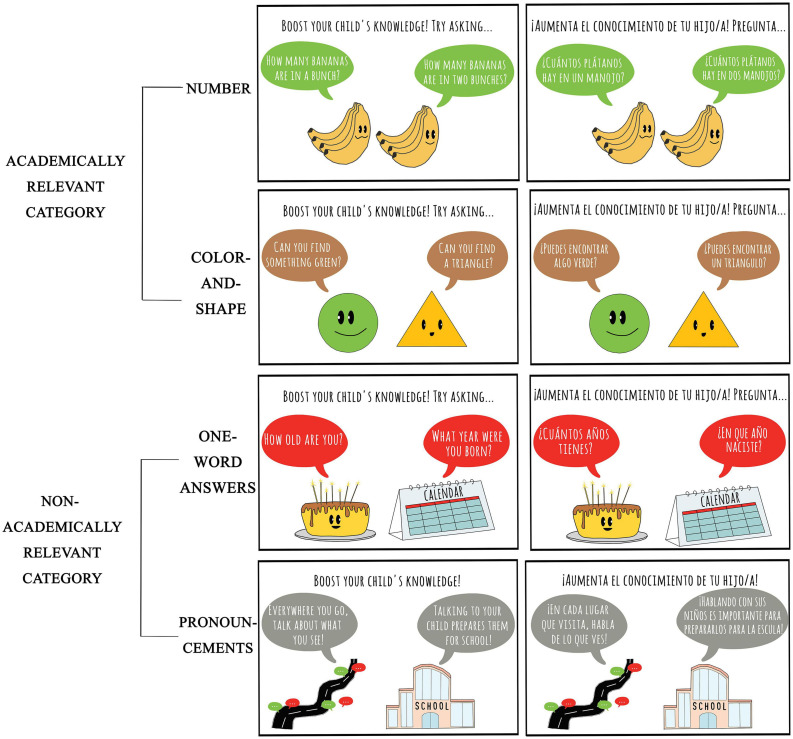
Samples of the academically-relevant (top two rows) and non-academically-relevant (bottom two rows) signs. The remaining two sets of signs used in the study can be found in the Supplementary Material.

The current study aims to build upon these two successful studies. First, we will describe our study and then highlight the ways in which it is distinct from the previous studies. We examined whether displaying signs in an everyday environment could increase the *quantity* and *quality* of caregiver-child conversation and whether there was flexibility in the content of the conversation based on the questions on the signs. We observed families in a food pantry, a volunteer-run facility where people who are food-insecure can select food from available donations. This particular food pantry has two or three volunteers stationed in each aisle to greet the clients and restock the shelves as needed. Each client takes a shopping cart at the entrance and they push the cart through the aisles in a single-file line that winds through all aisles of the pantry. Approximately 25% of the clients have children with them when they visit the food pantry. The observers worked as volunteers in the aisles. Each family was observed up to four times across different aisles in the food pantry. In the baseline condition where no signs were displayed, the observer would greet the client and, if they had a child in the target age range, they would observe the caregiver-child conversation while the family passed through the aisle. After the family left the aisle, the observer would record notes about the characteristics of the conversation. In the condition where signs were displayed, the only difference was that the observer would greet the client and point out that there were signs for children. For examples of the sign content, see Figure 1.

Our study is different from Ridge et al. (2015) and Hanner et al. (2019) in terms of the setting, the use of prompting, the sample, and the design. First, the setting is different in that we examined whether the phenomenon would generalize to a new everyday environment, in this case, a food pantry. The context of this food pantry is different from a grocery store in that every client was greeted as they entered an aisle and they were offered various food options by volunteers. In addition, this context allowed us to prompt attention to the signs in a naturalistic manner. The rationale for this came from studies that discuss the positive effects of providing caregivers specific prompts that result in children’s learning. Previous research has demonstrated that short interventions using prompts can provide caregivers with the necessary scaffolding to incorporate critical number, color and shape language into their conversations with children. In addition, these prompts can help caregivers tailor their conversations to their children’s interests and preferences in informal learning contexts such as homes and museums (Vandermaas-Peeler et al., 2012a,b; Haden et al., 2014; Jant et al., 2014; Polinsky et al., 2017; Braham et al., 2018). Second, the sample in our study is different because we used a comprehensive sample instead of a convenience sample. Every family with a child that passed through the aisles of the food pantry on those particular days was observed at least once. Lastly, our design was different because we were able to observe each family multiple times. In the baseline condition with no signs, we primarily observed the *quantity* of the caregiver-child conversation in four different aisles. In the signs-up condition, we observed the *quantity* of caregiver-child conversation. Additionally, we had two different categories of signs within the signs-up condition: academically-relevant and non-academically-relevant. The academically-relevant signs were similar to Hanner et al.’s (2019) prompts about math, although we added questions about colors and shapes, too. The non-academically-relevant signs served as a control to ensure that any observed differences in the number, color or shape talk were a result of the academically-relevant prompts and not merely a result of posting signs. Within the signs-up condition, the number, color and shape talk between caregivers and children was observed for both academically-relevant and non-academically-relevant signs to indicate the *quality* of the conversation.

To clarify the difference between our two categories, examples of the academically-relevant category include: “How many bananas are in a bunch? How many bananas are in two bunches?” or “Can you find a triangle? Can you find something green?” The questions on these signs were adapted from the literature on the strong positive association between math and spatial talk and children’s academic outcomes (Levine et al., 2010; Gunderson and Levine, 2011; Fisher et al., 2013; Verdine et al., 2014a; Resnick et al., 2016). The second category of signs, non-academically-relevant signs, asked simple factual questions with one-word answers, or consisted of pronouncements which are broad statements that informed caregivers about the benefits of talking to their children. A few examples include: “How old are you? What year is it?” or “Everywhere you go, talk about what you see!”

We had three predictions: first, there will be a higher *quantity* of conversation between caregivers and children who are exposed to the condition with signs compared to the baseline condition with no signs; second, there will be a higher *quality* of conversational content when caregivers and children are exposed to the academically-relevant signs compared to the non-academically-relevant signs; third, caregivers will be flexible in tailoring the content of their conversation, in that they would be equally likely to engage in conversations about academically-relevant, as well as non-academically-relevant signs.

## Materials and Methods

### Participants

We observed a total of 212 families. In this context, we define a family as consisting of at least one adult and one child estimated to be between 2 to 10 years of age. A total of 132 families were observed during the signs-up condition and 80 families were observed during the baseline condition with no signs. Approximately half of the families we observed had a child who appeared to be between the ages of 2 to 5 (*n* = 107) and the rest appeared to be between the ages of 6 to 10 years (*n* = 93). The ages of children in the remaining 12 families were not recorded. The vast majority of target adults were female (89%). The target children were 55% female, 43% male and the remaining 2% were not recorded. Demographic information for our sample is included in the Supplementary Material. This information is approximate because it was based on visual appearance and summarized according to the most common assessment made by all the observers.

In our sample, we observed that approximately 56% of families spoke only English, 30% spoke only Spanish, another 8% spoke both Spanish and English. The language(s) spoken by the remaining 6% of families was not recorded. Data were originally collected from 221 families, however, seven families were excluded for the following reasons: Two families (less than 1%) spoke a language other than English or Spanish and were eliminated from the final sample because the coders could not accurately record the characteristics of the conversation. Two families were excluded because the observers independently recorded the valence of the target child’s conversation as negative or very negative (i.e., crying, screaming behaviors) across multiple aisles, rendering engagement with the signs and conversational coding impossible. Five additional families were also excluded because the observers recorded the target child’s age to be 1 year and might have been potentially too young to benefit from the intervention.

The study was exempt from IRB review under category 2 because we observed public behavior. All families were identified by the number on the cart that they pushed through the food pantry. The demographic information was observational in nature and the data do not contain any identifiable variables. Consequently, we were not required to collect informed consent or debrief participants. We obtained written permission by the administration of the food pantry to conduct our study on their premises.

A minimum stopping rule of *n* = 180 was chosen based on similar prior research studies conducted in a grocery store (Ridge et al., 2015; Hanner et al., 2019). However, since the study design of these previous studies was significantly different from our study design, we ran a sensitivity analysis on our between-subjects variable (no-signs vs. signs-up) using GPower 3.1.9.6 (Faul et al., 2007). This sensitivity analysis computed the required effect size and was based on a chi-squared goodness-of-fit test, with an *α* = 0.05, power (1–*β*) = 0.95, total sample size (*N*) of 212, and *df* = 2. We obtained a resulting critical χ^2^ value of 5.99 and an effect size (*w*) of 0.27 (the smallest effect that could be reliably detected given the *α*, power, total sample size, degrees of freedom, and design/assumptions of the study). These resulting values are similar to sensitivity analyses conducted on the results of Hanner et al. (2019) [critical χ^2^ value of 5.99 and an effect size (*w*) of 0.29 obtained by using an *α* = 0.05, power (1–*β*) = 0.95, total sample size (*N*) of 179, and *df* = 2]. In addition, a sensitivity analysis was conducted on our within-subjects variable (academically-relevant vs. non-academically-relevant signs) using GPower. The sensitivity analysis was based on a Poisson regression with *α* = 0.05, power (1–*β*) = 0.95, total sample size (*N*) of 132, base rate (*β_0_*) of 0.01, and a binomial distribution of the predictor. We obtained a critical *z* value of 1.64 with a Exp(*β_1_*) value of 4.76 indicating the smallest effect that could be reliably detected given the above parameters.

### Procedure

All observations were conducted during the weekly distribution hours that occurred on Mondays and Thursdays between 9:30 am and 2 pm at a food pantry located in a suburb of a major metropolitan city in the United States. The data were collected over the course of five consecutive distribution days. The first and the fourth days consisted of the baseline condition with no signs and the remaining 3 days of observations comprised the signs-up condition when signs were displayed. A total of 80 families were observed during the baseline condition (45 and 35 families observed on each day, respectively). A total of 132 families were observed across the 3 days of the signs-up condition (44, 46, and 42 families observed on each day, respectively). Families visited the food pantry as often as once a week, but typically came only once a month, making it highly likely that data collected on different days was entirely between-subjects. Due to the observational nature of the study, we were unable to record the number of times a specific family visited the food pantry during our observation period. However, one benefit of our study design was that the signs were different on each day that we collected data. On the remote chance that a family was observed twice across observation days during our study, they did not see the same signs.

As mentioned above, this particular food pantry functions by taking food donations from local businesses and distributing them to people in need during specific hours twice a week. The distribution days are staffed by local volunteers who greet the clients in each aisle, offer specified quantities of each product, and restock the shelves as needed. There are usually two or three volunteers stationed in each aisle. The context of this food pantry was one where small talk among the volunteers and the families was the norm. Families answer many questions posed by the volunteers. For instance, volunteers often asked the adults questions like “Do you want a bag of lentils?”, “Would you like a box of this cereal or that one?”, or “We have pancake mix today too! Would you like a box?”. Volunteers regularly engaged with all the children passing through the aisles by asking for high-fives, checking in on their schooling, telling them that they were wearing cool shirts, and making such small-talk. Therefore, drawing the family’s attention to the signs (when displayed) with a statement like “there are signs to look at today!” was not out of the ordinary. Our observers worked primarily as volunteers because only about a quarter of the clients had children with them when they came through the food pantry.

Each client is given a shopping cart at the entrance to the food pantry. They move through all the aisles in a single-file line at a slow but steady pace. In both conditions, when a client entered the aisle, the observer would greet them, offer the contents on the shelf, and engage in small talk as the line progressed through the aisle, as is standard for volunteers in this food pantry. In the baseline condition, if the client had a child with them, the observer would observe the conversation between the caregiver and child in addition to greeting them and offering food. After the family left the aisle, the observer would write down the details of the conversation on a coding sheet. In the signs-up condition, the only difference was that, if the client that had a child with them, the observer would also tell the caregiver that there were signs up to entertain the children.

In both the baseline condition with no signs and the signs-up conditions, the observers were located in four different aisles—dry goods, freezer, bread, and produce. This means that a single family was observed four times during their time at the food pantry. Due to the observational nature of the study, it was not possible for the observers to be unaware of the contents of the sign in their aisle. However, our critical comparisons depend on codes made by independent observers who were unaware of the caregiver-child conversation in the other aisles. Across the five observation days, the observers varied across sign conditions (baseline and signs-up) and aisle locations. All observers were trained for approximately 6 hours in observation coding techniques prior to data collection. All observers were fluent in English and half of the observers who were also fluent in Spanish coded conversations of families that spoke Spanish. At least one or two observers (out of four observers) present on each observation day were fluent in Spanish.

The study consisted of a mixed design with the between-subjects factor of signs condition (baseline with no signs or signs-up) and the within-subjects factor of sign type (academically-relevant and non-academically-relevant). Within the academically-relevant signs, there were two levels: number and color/shape. Within the non-academically-relevant signs, there were two levels: one-word answers and pronouncements. Finally, there were three sets of signs so that we could counterbalance the location and type of each sign. For example, the number questions might be in the freezer aisle on day one (“How many eggs are in a dozen?), in the bread section on day two (“How many slices of bread are in a sandwich?”), and the produce aisle on day three (“How many bananas are in a bunch?”). The Supplementary Material contains a table with the complete list of prompts used in each aisle on the three signs-up days. The counterbalancing across days/aisles ensured that no particular question was responsible for the differences in our within-subject factors.

When families had more than one child in the target age range, the observer chose a single child as the target child based on the following predetermined rule: All the shopping carts in the food pantry were numbered. If the cart was an odd number, the target child was the older child (or the oldest in the rare case of three or more children). If the cart was an even number, the target child was the younger child (or youngest in the rare case of three or more children). This rule allowed multiple observers across different aisles to observe the same child unobtrusively.

Two observers simultaneously observed and double-coded 28 of the 219 families to establish reliability. These double-coded observations were evenly distributed across baseline and signs-up conditions, as well as across the four aisles of the food pantry. The observers had 87% inter-rater joint probability agreement on double-coded variables related to the *quantity* and *quality* of caregiver-child conversations.

### Coding

Our coding scheme was modeled after the methods of Hanner et al. (2019). We coded for the following variables: the valence of the overall caregiver-child interaction, the number of conversational turns, whether specific number, color, and shape talk was discussed, and observed demographics. Coding of these conversations was done in the moment and not transcribed.

*Quantity* of conversation is indicated by the number of conversational turns within a family. Conversational turns were defined as the number of times the adults and children in a group took turns to speak to the target child, or the target child spoke to one of the family members. A turn consisted of a single word, sentence, or a few sentences that were not interrupted or broken by another speaker. It included verbal comments and non-verbal gestures, like responsive head nods or pointing, that was directed toward or originated from the target child. We did not include conversational turns in situations where the adults in the group or children outside the targeted age range were conversing among themselves and were not engaging the target child. The number of conversational turns was coded in the following ranges: 0, 1, 2, 3–5, 6–9, 10–15, 16–20, and 20+. These ranges were collapsed into the following three bins during analyses: 0–5, 6–15, and 16+. Since chi-squared analyses with either set of bins were significant, we chose to collapse conversational turns into three bins for simplicity and alignment with Ridge et al. (2015) who also used three bins.

*Quality* of conversation is operationalized by whether the families incorporated academically-relevant content such as numbers, colors, and shapes into their conversation during the observation period. To measure this variable, the observers marked the sheet when the family engaged in conversations related to the following six domains: used numbers, elicited numbers, counted numbers, pointed to colors or shapes, used color or shape words, and elicited color or shape words. These domains were binary coded (present vs. absent) when the target child or any adult(s) within the family engaged in any of these behaviors at least once. A behavior was coded with a score of 1 if it was present and 0 if it was absent. This score ranged from 0 to 6. For example, if a child saw the sign: “How many bananas are in a bunch? How many bananas are in two bunches?,” counted and answered that there were five bananas in a bunch and 10 bananas in two bunches, this would result in a score of 2, with 1 point for using numbers and 1 point for counting. In contrast, a child who saw the sign: “Everywhere you go talk about what you see” may have talked about products in the aisle like the cereal box. In our coding scheme, this would be scored as zero unless the child or caregiver mentioned the cereal box was yellow or the shape of the cereal box was rectangular.

### Analysis Plan

To assess whether the presence of signs increased the *quantity* of conversation between caregivers and children, we performed a chi-squared analysis on the number of conversational turns across the between-subjects variable of signs condition (baseline with no-signs vs. signs-up). Next, to analyze whether there was a difference in the *quality* of caregiver-child conversation, we conducted a mixed-effects Poisson regression. This type of analysis was used because our dependent variable was a count variable of the amount of number, color and shape talk discussed by each family during the length of the observation and it followed a Poisson distribution. Finally, we examined whether the effect was carried by a specific type of sign. We conducted mixed-effects Poisson regressions to measure differences in the number, color and shape talk produced by families across the four different types of signs. To account for other variables that might have influenced the number, color and shape talk discussed by caregivers and children, we included the target child’s gender and age as fixed effects and random intercepts by family unit in all the Poisson regression models. All categorical variables were coded as indicator variables during analysis and missing observations were omitted by the mixed-effects Poisson regression models. All the analyses and visualizations were performed in R Studio (R Core Team, 2020) using the “stats” and “ggplot2” packages from RStudio, and the “lme4” package (Bates et al., 2014). A fully reproducible repository hosting the coding sheet, data, and analyses can be found at: https://github.com/apoorvashivaram/foodpantry.

## Results

**FIGURE 2:**
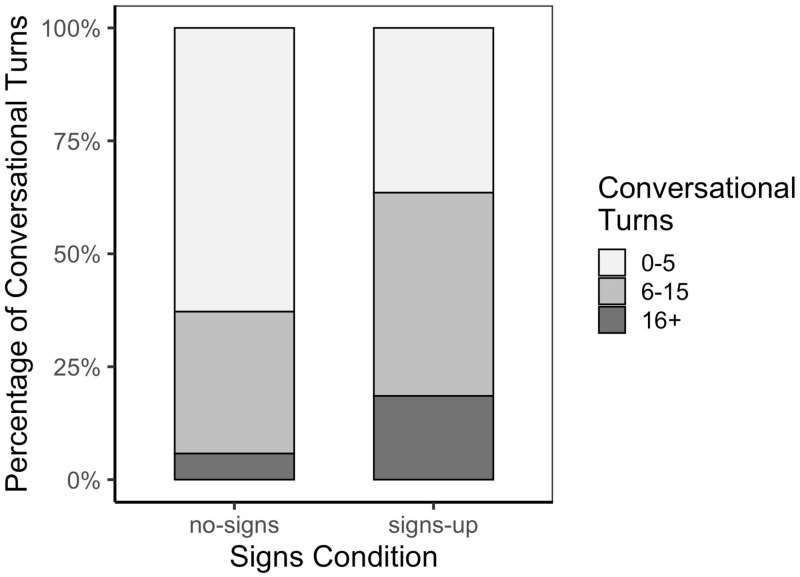
Percentage of conversational turns across the baseline with no-signs versus the signs-up conditions.

As show in Figure 2, there was a significantly higher number of conversational turns in the signs-up compared to the baseline condition with no signs, as indicated by a Pearson’s chi-squared test, χ^2^(2) = 44.13, *p* < 0.001. For the baseline condition, the majority of families (63%) had fewer than five conversational turns, 31% had 6–15 turns, and only 6% had 16 + turns. This pattern was reversed for the signs-up condition with the majority of families in the latter two bins—36% of families had conversations with 0–5 turns, 45% had 6–15 turns and 19% had 16 + turns.

**FIGURE 3:**
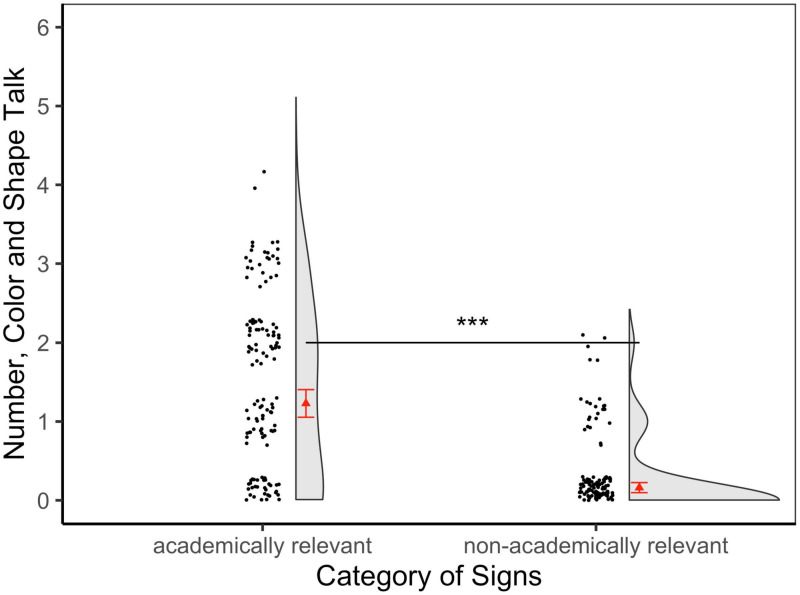
Number, color, and shape talk produced by families across the academically-relevant and non-academically-relevant sign categories. This score ranged from 0 to 6. The red triangle represents the mean and the red whiskers are standard error bars. The black dots represent the individual data points in the distribution and the half-violin plot represents the density of the distribution at different levels of the dependent variable. *** *p* < 0.001.

Our next analysis revealed that there was a significant difference in conversational content based on the category of sign. The academically-relevant signs had an average of 1.23 target domains discussed (*SD* = 1.14; Range: 0–4) compared to the non-academically-relevant signs that had an average of 0.16 (*SD* = 0.44; Range: 0–2) (see Figure 3).

The number, color and shape talk based on the type of signs (academically-relevant or non-academically-relevant; with non-academically-relevant as the reference group) was also predicted by a mixed-effects Poisson regression with child’s gender and age as fixed effects and random intercepts by family unit. Particularly notable is that the number, color and shape talk increased by a factor of 6.72 compared to non-academically-relevant signs, when accounting for child’s gender and age as fixed effects (see Table 1). This value of 6.72 was obtained by exponentiating the estimate for academically-relevant signs (*β* = 1.905) since every unit increase in the predictor variable “type of signs” (that is, from non-academically-relevant to academically-relevant signs) has a multiplicative effect of *exp*(*β*) on the mean of the dependent variable (here, number, color and shape talk). Approximately 63% of families observed near the academically-relevant signs discussed number, color, or shape talk compared to only 14% near the non-academically-relevant signs. Taken together, these results indicate that, after attention was directed to both categories of signs, the academically-relevant signs led to more number, color and shape talk.

**TABLE 1:** Results of the fixed-effects factors of the Poisson regression predicting the number, color and shape talk across the two signs-up categories.

**Predictor**	**Number, shape and color talk**
Intercept	−2.184*** (0.310)
Academically-relevant signs	1.905*** (0.219)
Child’s gender—Male	−0.173 (0.160)
Target child’s age	0.086* (0.035)
*N*	275
logLik	−274.614
AIC	559.229

The reference group for this analysis is the non-academically-relevant signs, with a target child who was a 2-year-old female. Values in each cell are estimates and their standard errors. * indicates p < 0.05 and *** indicates p < 0.001.

**FIGURE 4:**
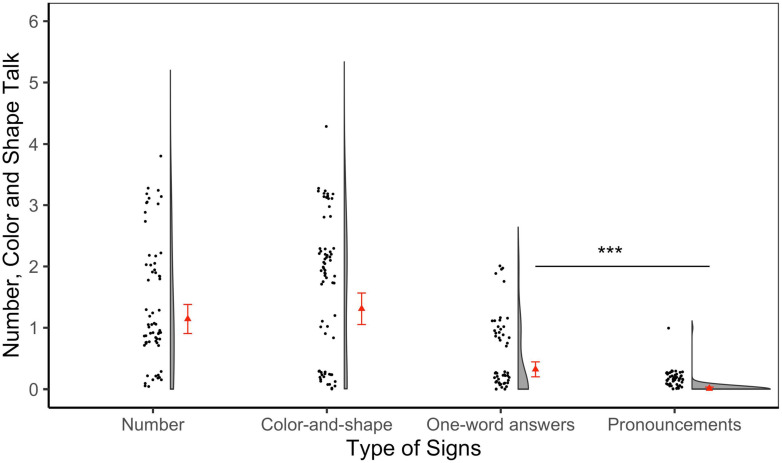
Number, color, and shape talk across the four types of signs. This score ranged from 0 to 6. The red triangle represents the mean and the red whiskers are standard error bars. The black dots represent the individual data points in the distribution and the half-violin plot represents the density of the distribution at different levels of the dependent variable. *** *p* < 0.001.

Finally, our results indicate that the differences between the academically-relevant and non-academically-relevant categories are not carried by any particular type of sign within the academically-relevant category (Number: *M* = 1.14, *SD* = 1.06, Range: 0–4; Color/shape: *M* = 1.31, *SD* = 1.20, Range: 0–4). However, there were differences within the non-academically-relevant category. The one-word mean was significantly higher (*M* = 0.32, *SD* = 0.58, Range: 0–2) than pronouncements (*M* = 0.01, *SD* = 0.10, Range: 0–1); however, both means were low and the variance for pronouncements was small (see Figure 4).

To examine differences in type of talk produced within families across the four types of signs, three mixed-effects Poisson regressions were conducted while controlling for the type of sign, with child’s gender and age as fixed effects and with random intercepts by family unit. There was no significant difference in number, color and shape talk between the number and color/shape signs (*β* = 0.26, *SE* = 0.16, *p* = 0.10). However, number signs prompted significantly higher talk about the target domains than one-word answers signs (*β* = −1.10, *SE* = 0.24, *p* < 0.001) and pronouncements signs (*β* = −4.29, *SE* = 1.01, *p* < 0.001). Color/shape signs prompted significantly higher talk about the target domains than both one-word answers (*β* = −1.36, *SE* = 0.23, *p* < 0.001) and pronouncements signs (*β* = −4.55, *SE* = 1.01, *p* < 0.001). Finally, one-word answers signs prompted significantly higher talk about the target domains than pronouncements (*β* = −3.18, *SE* = 1.02, *p* = 0.002) (see Supplementary Material for the regression tables).

## Discussion

The main goal of this study was to examine whether a brief intervention could change the conversation between caregivers and their children. We found that there were significantly more conversational turns between caregivers and children when their attention was prompted to the signs during the signs-up condition compared to the baseline condition with no-signs. We interpret these findings as evidence that a brief intervention and prompting families’ attention to the signs can change the *quantity* of conversations in an everyday environment of getting food at a food pantry. Our second goal was to investigate whether the *quality* of conversations was influenced by the contents of the sign. We found that academically-relevant signs encouraged number, color and shape talk compared to non-academically-relevant signs, despite the fact that families were prompted to attend to both categories of signs equally. Our third goal was to investigate whether caregivers were able to tailor the content of their conversation according to the type of sign displayed. By observing caregivers at several different time points, we found that they were equally adept at fostering different types of conversations. Moreover, these results are not due to specific types of questions since both number and color/shape questions on the signs were equally effective in prompting higher *quality* conversations, while one-word answers and pronouncements signs yielded significantly less number and color/shape talk. Together, this brief intervention of displaying signs and prompting families’ attention toward them increases the *quantity* and *quality* of caregiver-child conversations.

These results provide a conceptual replication of previous findings by Ridge et al. (2015) and Hanner et al. (2019) since the setting and procedure for our study was quite different from the previous work. Ridge et al. (2015) and Hanner et al. (2019) conducted their observations in grocery stores and it was left up to chance whether the families saw or read the signs. In contrast, we collected data in a food pantry where families who are food-insecure visit to receive donations. In this food pantry, all clients entered the aisles in a single-file line, were greeted, and families were also informed that the signs were for entertaining the children. Another difference was that Ridge et al. (2015) and Hanner et al. (2019) employed a convenience sampling technique whereas our sample included nearly all the families who visited the food pantry on the distribution days when the observations took place. Our findings align with previous research showing that brief interventions that promote math and spatial talk can encourage caregivers to engage their children in conversations (Siegler and Ramani, 2008; Hassinger-Das et al., 2020a). Additionally, Haden et al. (2014) have demonstrated that families who received prompts during a museum visit were significantly more likely to ask relevant questions and promote STEM-related conversations. Similarly, Braham et al. (2018) provide evidence that when parents talk to children in a grocery store, children’s spontaneous focus on number was significantly greater compared to when families discussed healthy eating concepts. Although there was no direct correlation between amount of math talk and children’s increases in their spontaneous focus on number, the study by Braham et al. (2018) provides sufficient causal evidence for a link between parent-child conversations and children’s increases in their spontaneous focus on number.

This study is a successful example of conducting ecologically valid research in an everyday environment. An important implication that can be drawn from these results is that stakeholders who are implementing future interventions in everyday contexts might benefit from specifically addressing the target outcomes they are interested in (e.g., conversations about academically-relevant content; increasing the *quantity* of conversation; entertainment). The best possible outcome is to design everyday environments by seeking input from those who frequent these everyday locations and to incorporate stakeholders who are educators or developmental scientists who could help design successful learning opportunities.

Yet, there are a few limitations to be considered in regards to the research design and context of this study. First, it is unclear whether short conversations about a variety of academically-relevant concepts or more in-depth conversation about one concept is the critical factor in promoting school readiness through conversations between caregivers and children. Most prior research has examined the effects of the amount of math or shape talk as given by the frequency of occurrence, but there are a few studies that have assessed the variability in the types of words being used in conversation (e.g., Eason and Ramani, 2020). The coding scheme for this study was designed to measure conversations about multiple academically-relevant concepts such as numbers, colors, and shapes. Future research can build on this coding scheme by distinctly coding for both breadth and depth of relevant topics and analyze whether one concept is more important than the other in promoting school readiness.

Second, a result of the naturalistic observational study design was that the observers were not blind to the conditions. All caregiver-child conversations were coded in the moment, not transcribed, and thus, were also limited in the amount of detailed coding information the observers could obtain during the brief observations. To reduce the amount of bias introduced into the coding, we had independent observers positioned in four aisles and the individual observers differed by days. Future studies could design double-blind data collection processes by training independent volunteers to observe the conversations.

More broadly, this study raises possible avenues for future research. One question pertains to the optimal dosage: how much exposure to signage with academically-relevant goals is necessary before the conversational benefits generalize to other contexts? Would the context of the food pantry serve as a sufficient prime to prompt conversations about academically-relevant concepts the next time the families visit the food pantry? Or do the signs have to be displayed for several weeks to result in long-term benefits? Additionally, in the current study, we were unable to determine whether these conversations influenced children’s learning since our data are limited to the immediate context of the food pantry. To better understand the scope and generalization of these studies to other context, future research could send follow-up surveys to caregivers shortly after these brief interventions to track whether these effects extended to other everyday contexts and whether there is an increased awareness among family members about the importance of early learning.

In conclusion, our findings demonstrate that the presence of signs is associated with a greater number of conversational turns between caregivers and children and that the type of signs (specifically, academically-relevant signs) prompted conversations about number, color and shape talk. Together, these findings suggest that it is possible to implement brief interventions that can influence the *quantity* and *quality* of caregiver-child conversations in everyday contexts that can potentially promote academic achievement and school readiness.

## Data Availability Statement

The original contributions presented in the study are publicly available. This data can be found here: https://github.com/apoorvashivaram/foodpantry.

## Ethics Statement

Ethical review and approval was not required for the study on human participants in accordance with the local legislation and institutional requirements. Written informed consent from the participants’ legal guardian/next of kin was not required to participate in this study in accordance with the national legislation and the institutional requirements.

## Author Contributions

AS contributed to the conceptualization, study design, data collection, data analysis, visualization and interpretation, and writing, editing, and revising the manuscript. YC, EA, AF, RJ, LE, and SP were contributed to the study design, data collection, and review of the manuscript. ML contributed to the conceptualization, study design, and review of the manuscript. SH contributed to the conceptualization, study design, data collection and interpretation, and writing, editing, and revising the manuscript. All authors contributed to the article and approved the submitted version.

## Conflict of Interest

The authors declare that the research was conducted in the absence of any commercial or financial relationships that could be construed as a potential conflict of interest.
