# Supplementary material for: Brief Interventions Influence the Quantity and Quality of Caregiver-Child Conversations in an Everyday Context
Source: Front Psychol. 2021 Jun 16;12:645788. doi: 10.3389/fpsyg.2021.645788 (PMC8242245; doi:10.3389/fpsyg.2021.645788)
Supplement: Supplementary file 1 [file Data_Sheet_1.docx]

Supplementary Material

# Supplementary Data

The dataset used in this study and R scripts from data analyses can be found in the following GitHub repository: <https://github.com/apoorvashivaram/foodpantry>

# Supplementary Figures and Tables

## Supplementary Figures

**
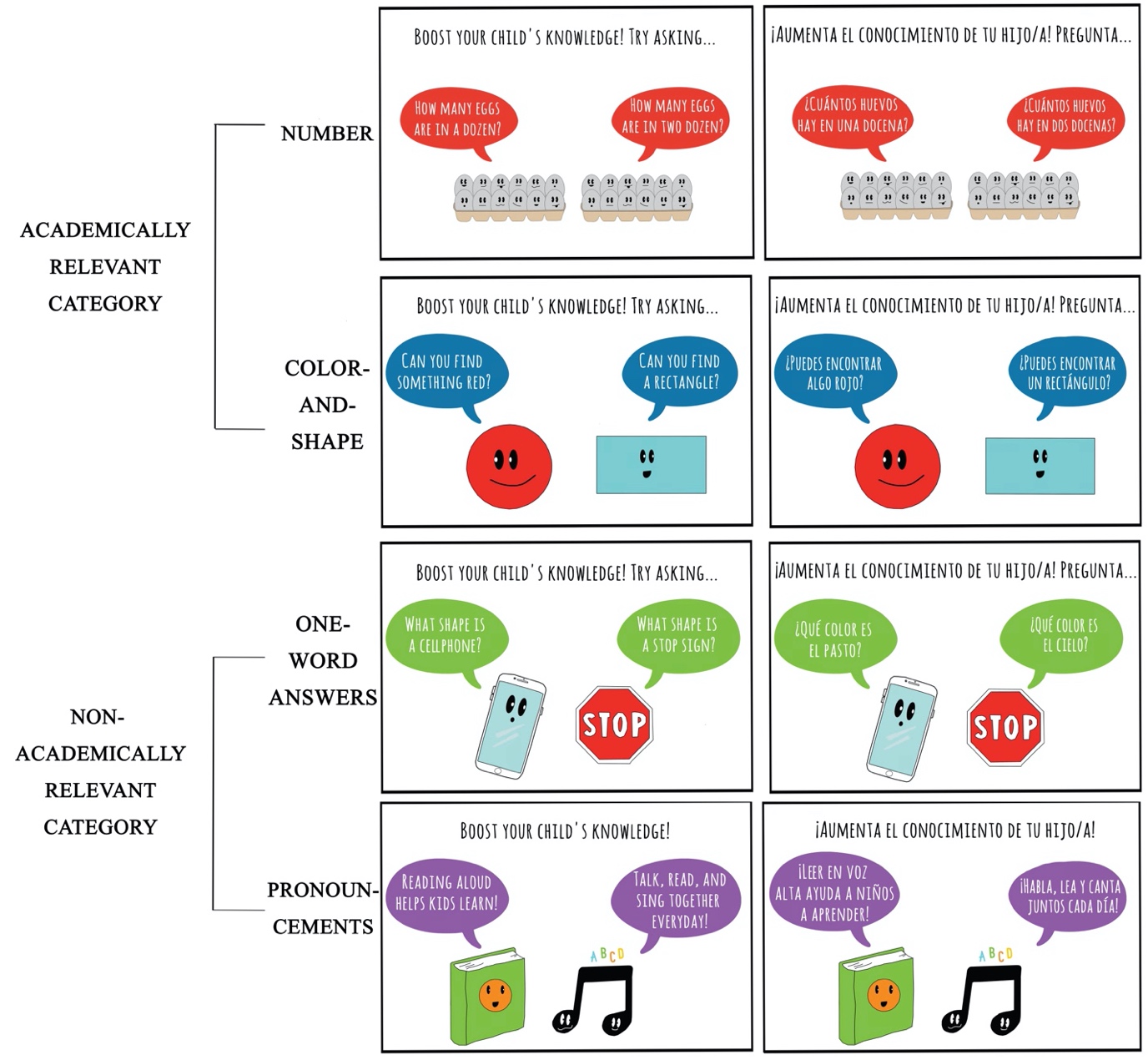
**

**Supplementary Figure 1.** Academically-relevant (top two rows) and non-academically-relevant (bottom two rows) signs that were displayed in the food pantry on the first day of the signs-up condition.

**
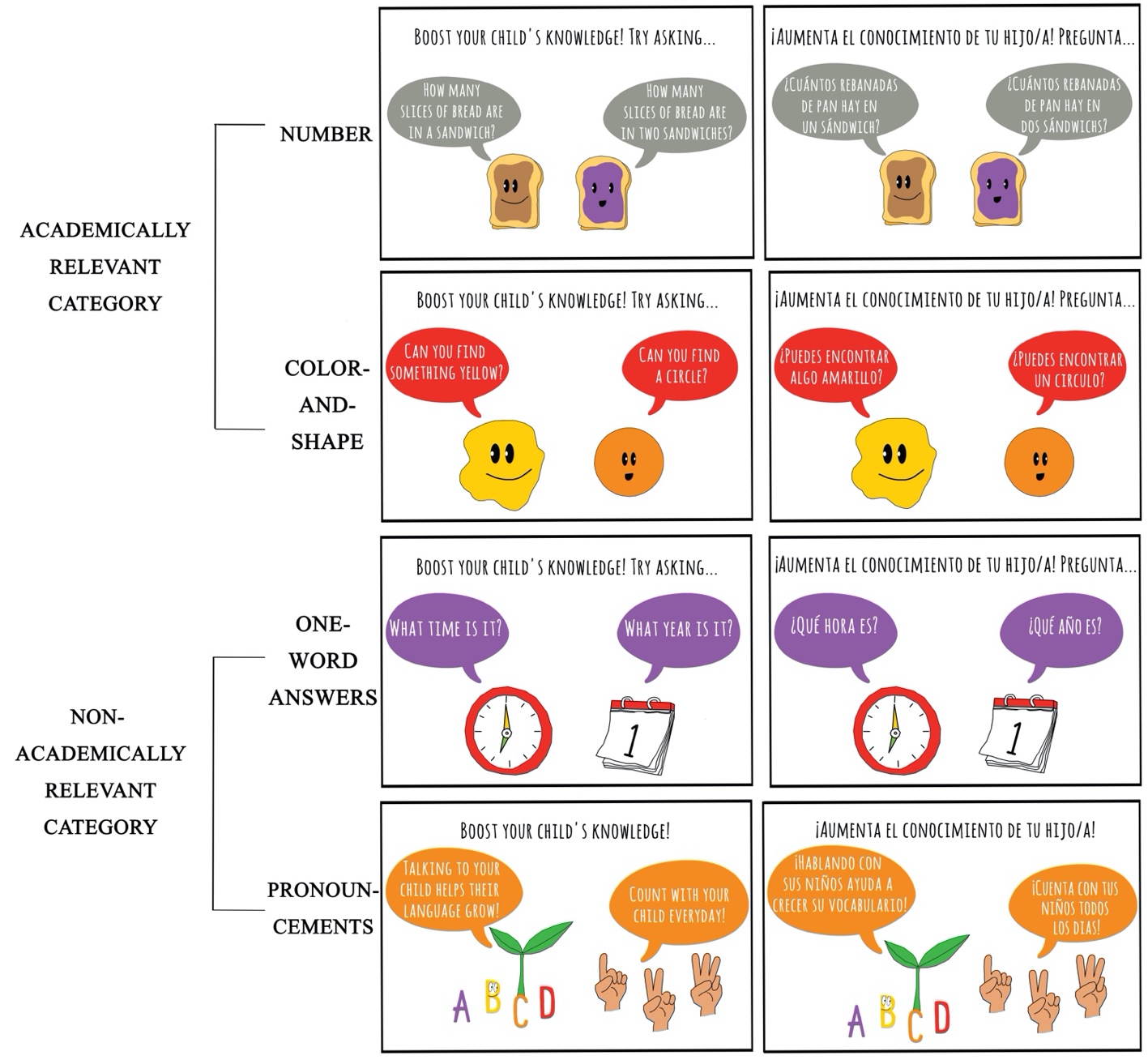
**

**Supplementary Figure 2.** Academically-relevant (top two rows) and non-academically-relevant (bottom two rows) signs that were displayed in the food pantry on the second day of the signs-up condition.

## Supplementary Tables

**Supplementary Table 1.** Observed demographics of the participants across all conditions, total *n* = 212. All demographic variables were judged based on visual appearance by the observers. These values were obtained by aggregating and finding a consensus among the demographics recorded by multiple independent observers. No information was collected from any of the participants.

| Variable | Overall | Signs-down | Signs-up |
| --- | --- | --- | --- |
| Group structure |  |  |  |
| One adult & one child | 100 | 27 | 73 |
| One adult & multiple children | 59 | 26 | 33 |
| Multiple adults & one child | 36 | 20 | 16 |
| Multiple adults & multiple children | 12 | 7 | 5 |
| Not recorded | 5 | - | 5 |
| Target adult gender |  |  |  |
| Female | 188 | 71 | 117 |
| Male | 18 | 9 | 9 |
| Not recorded | 6 | - | 6 |
| Target child gender |  |  |  |
| Female | 116 | 41 | 75 |
| Male | 91 | 39 | 52 |
| Not recorded | 5 | - | 5 |
| Target child age |  |  |  |
| Two | 13 | 6 | 7 |
| Three | 29 | 10 | 19 |
| Four | 32 | 11 | 21 |
| Five | 33 | 13 | 20 |
| Six | 24 | 8 | 16 |
| Seven | 27 | 12 | 15 |
| Eight | 20 | 9 | 11 |
| Nine | 8 | 2 | 6 |
| Ten | 14 | 8 | 6 |
| Not recorded | 12 | 1 | 11 |
| Family’s race/ethnicity |  |  |  |
| Asian | 13 | 4 | 9 |
| African American | 21 | 8 | 13 |
| Latinx | 144 | 54 | 90 |
| White | 26 | 12 | 14 |
| Multiracial | 2 | 2 | - |
| Not recorded | 6 | - | 6 |
| Language spoken |  |  |  |
| English | 118 | 44 | 74 |
| Spanish | 18 | 29 | 34 |
| English and Spanish | 63 | 5 | 13 |
| Not recorded | 13 | 2 | 11 |

**Supplementary Table 2.** The academically-relevant and non-academically-relevant signs in English and Spanish as given by the four types of signs (columns) sorted according to each day of the signs-up condition (rows).

|  | Active-learning signs | | Low-engagement Signs | |
| --- | --- | --- | --- | --- |
|  | Number | Color-and-shape | One-word answers | Pronouncements |
| Signs-up  Day 1 | How many eggs are in a dozen? How many eggs are in two dozen?  ¿Cuántos huevos hay en una docena? Cuántos huevos hay en dos docenas? | Can you find something red? Can you find a rectangle?  ¿Puedes encontrar algo rojo? ¿Puedes encontrar un rectángulo? | What shape is a cellphone? What shape is a stop sign?  ¿Qué es la forma de un celular? ¿Cuál es la figura de un señal de alto? | Reading aloud helps kids learn! Talk, read, and sing together every day!  ¡Leer en voz alta ayuda a niños a aprender! ¡Habla, lea y canta juntos cada día! |
|  | (Freezer) | (Bread) | (Produce) | (Dry goods) |
| Signs-up  Day 2 | How many slices of bread are in a sandwich? How many slices of bread are in two sandwiches?  ¿Cuántos rebanadas de pan hay en un sándwich? ¿Cuántos rebanadas de pan hay en dos sándwichs? | Can you find something yellow? Can you find a circle?  ¿Puedes encontrar algo amarillo? ¿Puedes encontrar un circulo? | What time is it? What year is it?  ¿Qué hora es? ¿Qué año es? | Talking to your child helps their language grow! Count with your child every day!  ¡Hablando con sus niños ayuda a crecer su vocabulario! ¡Cuenta con tus niños todos los dias! |
|  | (Bread) | (Produce) | (Dry goods) | (Freezer) |
| Signs-up  Day 3 | How many bananas are in a bunch? How many bananas are in two bunches?  ¿Cuántos plátanos hay en un manojo? ¿Cuántos plátanos hay en dos manojos? | Can you find something green? Can you find a triangle?  ¿Puedes encontrar algo verde? ¿Puedes encontrar un triangulo? | How old are you? What year were you born?  ¿Cuántos años tienes? ¿En que año naciste? | Everywhere you go, talk about what you see! Talking to your child prepares them for school!  ¡En cada lugar que visita, habla de lo que ves! ¡Hablando con sus niños es importante para prepararlos para la escula! |
|  | (Produce) | (Dry goods) | (Freezer) | (Bread) |

**Supplementary Table 3.** Results of the fixed-effects factors of the Poisson regression predicting number, color and shape talk across the four signs (number, color-and-shape, one-word answers, and pronouncements signs). The reference group for this analysis is observations from the number sign type, with a target child who was a 2-year-old female. Values in each cell are estimates and their standard errors. ** p* < 0.05; **** p* < 0.001.

| *Predictor* | *Number, color and shape talk* |
| --- | --- |
| Intercept | -0.556 * (0.265) |
| Color-and-shape (academically-relevant) | 0.261 (0.156) |
| One-word answers (non-academically-relevant) | -1.102 *** (0.240) |
| Pronouncements (non-academically-relevant) | -4.285 *** (1.007) |
| Child’s gender – Male | -0.152 (0.157) |
| Target child’s age | 0.110 ** (0.035) |
| *N* | 275 |
| logLik | -260.478 |
| AIC | 534.957 |

**Supplementary Table 4.** Results of the fixed-effects factors of the Poisson regression predicting the number, color and shape talk across the four signs (number, color-and-shape, one-word answers, and pronouncements signs). The reference group for this analysis is observations from the color-and-shape sign type, with a target child who was a 2-year-old female. Values in each cell are estimates and their standard errors. ** p* < 0.05; **** p* < 0.001.

| *Predictor* | *Number, color and shape talk* | |
| --- | --- | --- |
| Intercept | -0.295 (0.234) | |
| Number (academically-relevant) | -0.260 (0.156) | |
| One-word answers (non-academically-relevant) | -1.363 *** (0.233) | |
| Pronouncements (non-academically-relevant) | -4.546 *** (1.006) | |
| Child’s gender – Male | -0.152 (0.157) | |
| Target child’s age | 0.110 ** (0.035) | |
| *N* | 275 |  |
| logLik | -260.478 |  |
| AIC | 534.957 |  |

**Supplementary Table 5.** Results of the fixed-effects factors of the Poisson regression predicting the number, color and shape talk across the four signs (number, color-and-shape, one-word answers, and pronouncements signs). The reference group for this analysis is observations from the one-word answers sign type, with a target child who was a 2-year-old female. Values in each cell are estimates and their standard errors. ** p* < 0.05; **** p* < 0.001.

| *Predictor* | *Number, color and shape talk* | |
| --- | --- | --- |
| Intercept | -1.658 *** (0.305) | |
| Number (academically-relevant) | 1.141 *** (0.240) | |
| Color-and-shape (academically-relevant) | 1.363 *** (0.233) | |
| Pronouncements (non-academically-relevant) | -3.183 ** (1.022) | |
| Child’s gender – Male | -0.152 (0.157) | |
| Target child’s age | 0.110 ** (0.035) | |
| *N* | 275 |  |
| logLik | -260.478 |  |
| AIC | 534.957 |  |
